# The molecular, immune features, and risk score construction of intraductal papillary mucinous neoplasm patients

**DOI:** 10.3389/fmolb.2022.887887

**Published:** 2022-08-26

**Authors:** Xing Huang, Yipeng Feng, Dawei Ma, Hanlin Ding, Gaochao Dong, Yan Chen, Xiaochen Huang, Jingyuan Zhang, Xinyu Xu, Chen Chen

**Affiliations:** ^1^ Department of Pathology, Jiangsu Cancer Hospital & Jiangsu Institute of Cancer Research & the Affiliated Cancer Hospital of Nanjing Medical University, Nanjing, China; ^2^ Department of Thoracic Surgery, Nanjing Medical University Affiliated Cancer Hospital & Jiangsu Cancer Hospital & Jiangsu Institute of Cancer Research, Nanjing, China; ^3^ Jiangsu Key Laboratory of Molecular and Translational Cancer Research, Cancer Institute of Jiangsu Province, Nanjing, China; ^4^ The Fourth Clinical College of Nanjing Medical University, Nanjing, China; ^5^ Department of Oncology, Jiangsu Cancer Hospital & Jiangsu Institute of Cancer Research & the Affiliated Cancer Hospital of Nanjing Medical University, Nanjing, China

**Keywords:** intraductal papillary mucinous neoplasm, gene expression omnibus, The Cancer Genome Atlas Program, immunohistochemistry, microenvironment

## Abstract

Intraductal papillary mucinous neoplasm (IPMN) is a common pancreatic precancerous lesion, with increasing incidence in recent years. However, the mechanisms of IPMN progression into invasive cancer remain unclear. The mRNA expression data of IPMN/PAAD patients were extracted from the TCGA and GEO databases. First, based on GSE19650, we analyzed the molecular alterations, tumor stemness, immune landscape, and transcriptional regulation of IPMN progression. The results indicated that gene expression changed dramatically, specifically at the intraductal papillary-mucinous adenoma (IPMA) stage. Gene ontology (GO), Kyoto Encyclopedia of Genes and Genomes (KEGG), and Kyoto Encyclopedia of Genes and Genomes (GSEA) pathway analyses showed that glycoprotein-related, cell cycle, and P53 pathways displayed the most significant changes during progression. With IPMN progression, tumor stemness increased continuously, and KRAS, ERBB3, RUNX1, and ELF3 are essential driver genes affecting tumor stemness. Motif analysis suggested that KLF4 may be a specific transcription factor that regulates gene expression in the IPMA stage, while MYB and MYBL1 control gene expression in the IPMC and invasive stages, respectively. Then, GSE19650 and GSE71729 transcriptome data were combined to perform the least absolute shrinkage and selection operator (LASSO) method and Cox regression analysis to develop an 11-gene prediction model (KCNK1, FHL2, LAMC2, CDCA7, GPX3, C7, VIP, HBA1, BTG2, MT1E, and LYVE1) to predict the prognosis of pancreatic cancer patients. The reliability of the model was validated in the GSE71729 and TCGA databases. Finally, 11 additional IPMN patients treated in our hospital were included, and the immune microenvironment changes during IPMN progression were analyzed by immunohistochemistry (IHC). IHC results suggest that Myeloid-derived suppressor cells (MDSCs) and macrophages may be key in the formation of immunosuppressive microenvironment of IPMN progression. Our study deepens our understanding of IPMN progression, especially the changes in the immune microenvironment. The findings of this work may contribute to the development of new therapeutic strategies for IPMN.

## Introduction

Pancreatic cancer is one of the most malignant and aggressive human cancers worldwide (Siegel et al., 2021). The overall 5 years survival rate is estimated to be less than 10%, whereas the median survival rate is only 6 months (Siegel et al., 2021). Most patients lose the opportunity to have radical surgery because they are diagnosed at a late stage. Therefore, early diagnosis of pancreatic cancer is essential for improved prognosis (Singhi et al., 2019).

Pancreatic ductal adenocarcinoma (PDAC) is known to arise from different precursor lesions, namely, pancreatic intraepithelial neoplasia (PanIN), intraductal papillary mucinous neoplasm (IPMN), and mucinous cystic neoplasm (MCN) (Hruban et al., 2007). PanINs are microscopic papillary or flat, noninvasive epithelial neoplasms usually <5 mm in diameter confined to pancreatic ducts. Due to their small size, they are easily missed on examination. In contrast, IPMN is a perceptible intraductal mucinous tumor, usually with a diameter of >1 cm and a high imaging detection rate (Shi and Hruban, 2012). Similarly to the adenoma-carcinoma sequence in colorectal cancer (Leslie et al., 2002), IPMN begins as an intraductal papillary-mucinous adenoma (IPMA), progresses to an intraductal papillary-mucinous carcinoma (IPMC), and then eventually develops into an invasive adenocarcinoma (Malleo et al., 2015). The survival rate drops dramatically when infiltration occurs and can get as low as that of PDAC in some cases (Holmberg et al., 2021).

In recent years, the detection rate of IPMN has been increasing with the advancement of medical imaging technologies, such as magnetic resonance imaging (MRI), computed tomography (CT), and endoscopic ultrasound (EUS) (Klibansky et al., 2012). Current IPMN research mainly focuses on tissue genetics, tumor marker detection, and cyst fluid analysis, with encouraging results (Thornton et al., 2013; Aronsson et al., 2018; Carr et al., 2018). However, no studies have comprehensively dissected the molecular changes throughout IPMN carcinogenesis (from the normal duct to IPMA, IPMC and to invasive).

Therefore, this study explores the genomic characteristics of IPMNs based on-chip information from public databases and analyzes the key IPMN transition processes. In addition, immunohistochemical analysis of IPMN specimens was performed to explore immune cell subsets during IPMN progression to deepen the understanding of the IPMN microenvironment.

## Materials and methods

### Human tissue samples

Tumor tissue samples were obtained from IPMN patients who underwent surgical resection at Nanjing Medical University Affiliated Cancer Hospital from 2007 to 2018 (Table 1). The Internal Review Board of Nanjing Medical University Affiliated Cancer Hospital approved this study, and all patients provided written informed consent.

**TABLE 1:** Clinicopathological data from included analyzed IPMNs in Nanjing medical affiliated cancer hospital.

Case	Age	Gender	Location	Tumor size (cm)	CEA (ng/ml)	CA19-9 (U/ml)	Pathological diagnosis
Cas-01	59	Female	Body	2	1.2	9.36	IPMA
Cas-02	51	Male	Head	6	0.957	6.83	IPMC
Cas-03	67	Male	Body	1.5	4.93	11.66	IPMC
Cas-04	58	Female	Body	3.5	1.05	6.33	IPMA
Cas-05	72	Female	Body	1.5	3.53	125.9	IPMC/IC
Cas-06	62	Male	Body	4	5.64	151	IPMC
Cas-07	54	Female	Head	1.5	1.3	12.2	IPMA
Cas-08	68	Male	Head	1.5	1.08	7.12	IPMA
Cas-09	66	Female	Head	1.8	1.39	61.2	IPMC
Cas-10	65	Male	Body	2	5.72	1.5	IPMA
Cas-11	70	Male	Body	3.5	1.22	106	IPMC/IC

Cas, Case; IC, invasive cancer.

### Antibodies

The following antibodies were used in this study: anti-CD11b (ab52478, Abcam), anti-CD4 (4B12, DAKO), anti-CD8 (C8/144B, DAKO), anti-CD14 (ab183322, Abcam), anti-CD15 (Carb-3, DAKO), anti-CD33 (17425-1-AP, Proteintech), anti-HLA-DR (17221-1-AP, Proteintech), anti-CD20 (L26, DAKO), anti-FOXP3 (ab215206, Abcam), and anti-CD68 (PG-M1, DAKO).

### Immunohistochemistry analysis

The FFPE tissue samples were cut into 4 mm thin sections. Slides were incubated for 10 min in 0.3% hydrogen peroxide. Subsequently, sections were incubated overnight at 4°C with the primary antibody. Then, slides were incubated with a secondary antibody [either EnVision + System-HRP Labeled Polymer anti-rabbit or anti-mouse (Dako, Agilent, Santa Clara, United States)]. The average density (cells/mm^2^) of each lymphocyte subset was calculated from five 1 mm^2^ regions within each tumor to calculate its average density of positive cells (Reuben et al., 2017). IPMN was confirmed in all tissue samples by H&E staining, and the grade of dysplasia was evaluated by two experienced pathologists (ZJY and HX).

### Data collection

TCGA RNA sequence level 3 data of 182 PAAD samples and the related clinical information was obtained from UCSC_XENA (https://xena.ucsc.edu/) and the Cancer Genomics database (http://www.cbioportal.org/). Gene expression data were normalized by FPKM (fragments per kilobase million). Raw data from two microarray datasets, GSE19650 and GSE71729, were downloaded from the GEO database (https://www.ncbi.nlm.nih.gov/geo/). All data were publicly available (Hiraoka et al., 2011; Moffitt et al., 2015).

### Identification of differentially expressed genes and functional enrichment

Differently expressed genes (DEGs) between groups were identified using the “limma” R package (Ritchie et al., 2015), with the threshold of false discovery rate (FDR)-adjusted *p*-value < 0.05 and fold change (FC) > 1.5. The R package “ClusterProfiler” was applied for Gene Ontology (GO) and Kyoto Encyclopedia of Genes and Genomes (KEGG) pathway enrichment analyses. Gene set enrichment analysis (GSEA) was performed using the GSEA package (Subramanian et al., 2005).

### Stemness index production and driver gene expression

The Stemness indices were computed using the TCGA PanCanAtlas Stemness Project method (https://bioinformaticsfmrp.github.io/PanCanStem_Web/) (Malta et al., 2018) that was contraposed to mRNA expression (mRNAsi). Two hundred ninety-nine cancer driver genes from the PanCancer Project were downloaded, and their expression levels were compared between groups. Driver genes with FDR < 0.05 and FC > 1.5 were considered differentially expressed. Spearman’s rank correlation analysis was performed to evaluate the correlation between Stemness scores and the identified genes.

### Development of an eleven-gene risk scoring system for survival analysis

The combination of the two mRNA microarray datasets (GSE19650 and GSE71729; n = 379) was designated as the training dataset. The least absolute shrinkage and selection operator (LASSO) Cox regression model was performed by the R package “glmnet” to narrow down the consistent DEGs (coDEGs) to select the most useful prognostic markers, in which the training dataset was subsampled. The tuning parameter lambda was determined according to the expected generalization error estimated from leave-one-out cross-validation. Then a prognostic signature was constructed using the expression value of the identified genes and weighted by the regression coefficient. Finally, an eleven-gene signature was obtained under the tuning parameter lambda value, giving minimum mean cross-validated error. The risk score of the signature was then calculated using the risk score formula:

Risk score = (0.202* expression of MT1E) + (0.166* expression of KCNK1) + (0.155 * expression of GPX3) + (0.104* expression of LAMC2) + (0.017 * expression of HBA1) + (−0.005 * expression of LYVE1) + (−0.010 * expression of FHL2) + (−0.019* expression of C7) + (−0.038* expression of VIP) + (−0.079* expression of BTG2) + (−0.094* expression of CDCA7).

Kaplan–Meier survival analysis and the log-rank test were used to evaluate the prognostic differences between the two groups. Univariate and multivariate Cox proportional hazard regression analyses evaluated independent prognostic factors.

### Motif analysis

Homer (v4.11) was used to analyze the motifs of the promoter regions of DEGs (IPMA-upregulated, IPMC-upregulated, and invasive-upregulated DEGs, respectively) (Heinz et al., 2010). We selected 2000 bp upstream of the gene transcription initiation site as the gene’s promoter region. For each enriched motif, we extracted HOMER’s best matches to known motif transcription factors (TFs) to generate a list of putative TF factors. The enrichment results were visualized using the R package “ggseqlogo”.

### Immune cell infiltration assessment

To investigate the level of immune infiltration landscape, ssGSEA (Barbie et al., 2009) was performed in a sample according to the expression levels of immune cell-specific marker genes and recorded as ssGSEA score. The tumor purity, ESTIMATE score, stromal score, and immune score of each sample were calculated using the ESTIMATE algorithm (Yoshihara et al., 2013).

### Statistical analysis

R version 3.5.3 (http://www.R-project.org) and GraphPad Prism 8.0 (GraphPad Software, San Diego, CA, United States) were applied for statistical analysis and graph plotting. A 2-sided *p* value < 0.05 was statistically significant for Kaplan–Meier survival analysis and Mann–Whitney U test. The area under the ROC curve (AUC) was calculated to evaluate the prognostic performance of the identified signature. Univariate and multivariate Cox regression analyses were conducted to determine the prognostic performance of risk factors.

## Results

### Changes in biological functions during IPMN tumorigenesis

RNA-seq data and related clinical data were collected from 22 IPMN samples from the GEO dataset (GSE19650, including 7 normal main pancreatic ducts (normal), 6 intraductal papillary-mucinous adenomas (IPMAs), 6 intraductal papillary-mucinous carcinomas (IPMCs), and 3 invasive carcinomas arising from IPMNs).

**FIGURE 1:**
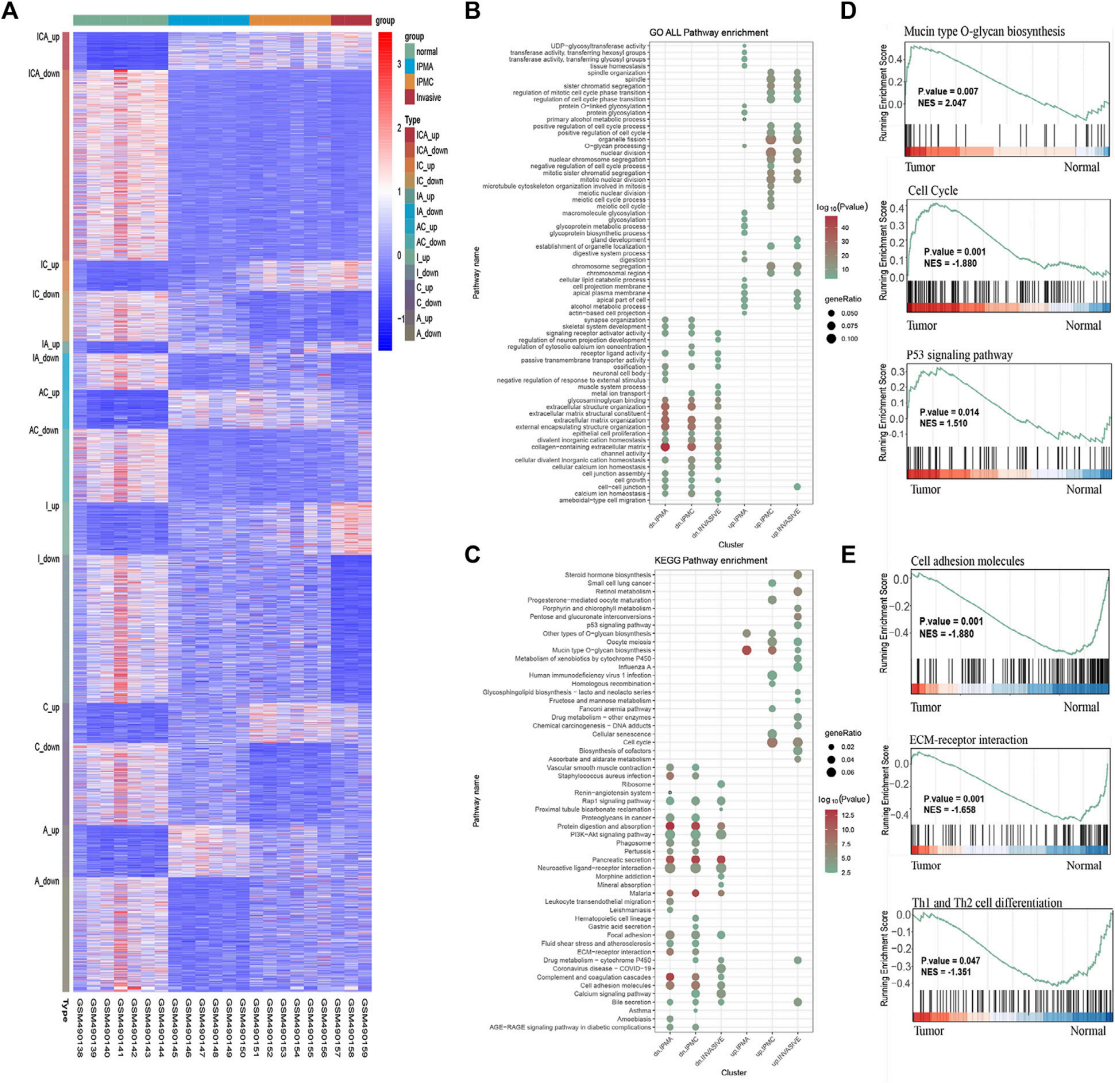
DEGs, GO, KEGG, and GSEA enrichment results. **(A)** Heatmap of DEGs in Normal, IPMA, IPMC and invasive samples. Up, up-regulated DEGs; down, down-regulated DEGs; ICA, IPMA, IPMC, and invasive common DEGs; IC, IPMC and invasive common DEGs; IA, IPMA and invasive common DEGs; AC, IPMA and IPMC common DEGs; I, C, and A invasive-, IPMC-, and IPMA-specific DEGs, respectively. **(B)** GO analysis in terms of biological processes; **(C)** KEGG enrichment of DEGs. **(D,E)** GSEA enrichment of DEGs.

Through differential gene expression analyses, we found many DEGs between IPMA, IPMC, INVASIVE, and normal duct (Figure 1A). Most of the IPMC DEGs and INVASIVE DEGs were already present in the IPMA. DEGs, indicating cancer-like genetic alterations in IPMA are shown in Figure 1A and Supplementary Figure S1.

To identify biological behavior changes during IPMN tumorigenesis, GO, KEGG, and GSEA enrichment analyses were performed. GO analysis showed that glycoproteins metabolic process, glycoproteins biosynthetic process, and glycosylation were significantly enriched in the IPMA group (Figure 1B). Contrary, in the IPMC and INVASIVE groups, the cell cycle and nuclear division were the most significantly changed biological processes (Figure 1B). KEGG and GSEA pathway analysis revealed that mucin-type O-glycan biosynthesis, cell cycle, and the P53 signaling pathway were the most significantly enriched pathways (Figures 1C,D). Pathways related to cell adhesion molecules, ECM-receptor interaction, and Th1/Th2 cell differentiation were the most significantly downregulated during IPMN progression (Figure 1E).

### The emergence of cancer-like changes from IPMN to INVASIVE

**FIGURE 2:**
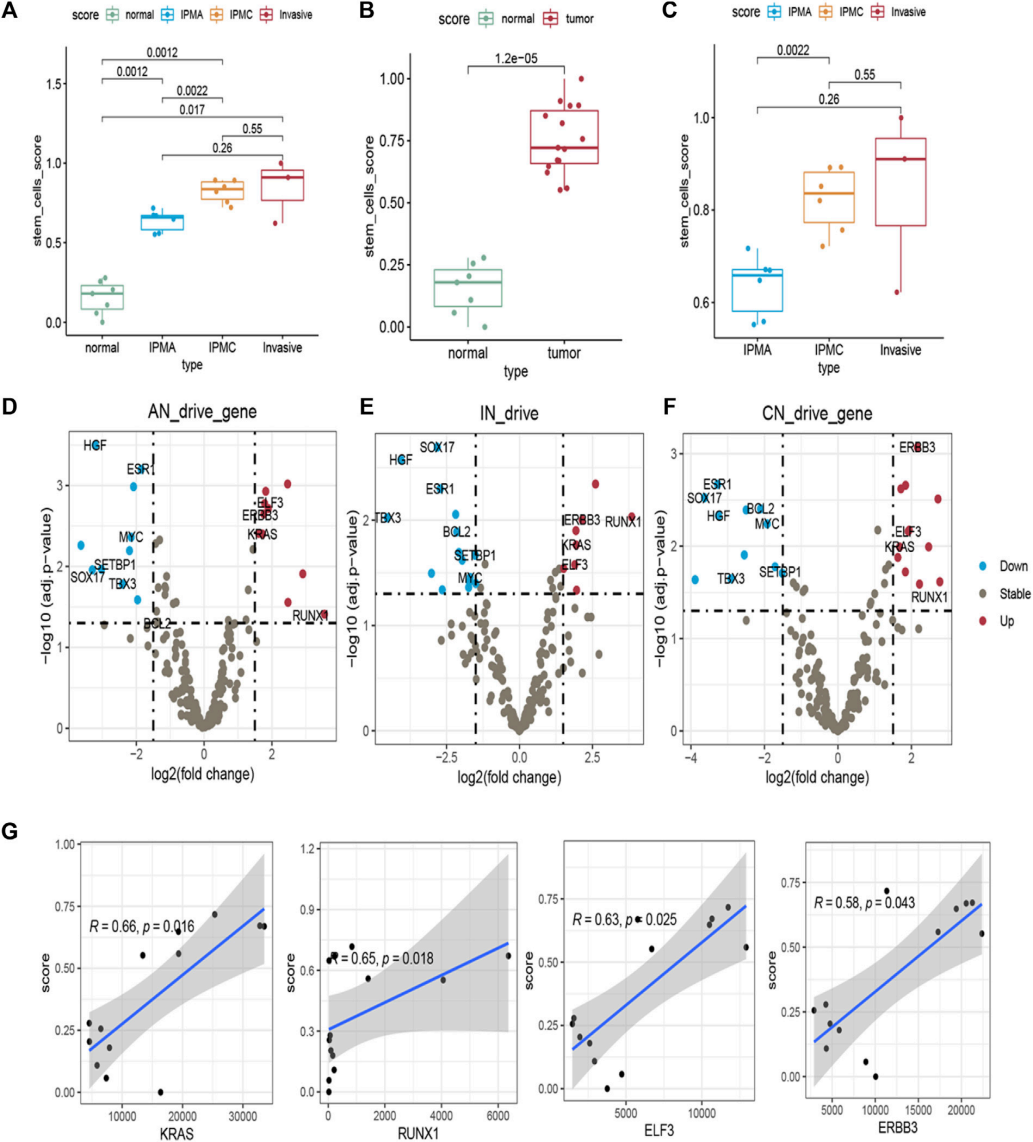
Stemness and expression of 299 pan-cancer driver genes in different disease stage tissues. **(A–C)** Stem scores of different samples. **(D–F)** Differential expression of 299 pan-cancer driver genes in **(D)** IPMA, **(E)** invasive, and **(F)** IPMC relative to Normal samples. Driver genes with *p* < 0.05 and fold-change >1.5 were identified as up driver genes (red dots). Driver genes with *p* < 0.05 and fold-change <1.5 were identified as down driver genes (blue dots). Other genes were assumed as unchanged driver genes (grey dots). **(G)** Correlation of stem scores and four consistent changed driver genes in IPMA, IPMC, and invasive samples.

To further explore functional changes during IPMN tumorigenesis, we characterized the stem cell-like features of different stages by stem score. The stem scores of IPMA, IPMC, and INVASIVE were significantly higher than those of normal (*p* < 0.05) (Figures 2A,C). Furthermore, the stem cell score showed a significant increasing trend from normal to INVASIVE (Figures 2A,B). Although the median stem cell score of INVASIVE was higher than that of IPMC, no significant differences were found (*p* > 0.05) (Figure 2B). These results indicate that some basic IPMA biological characteristics have changed at the molecular level, which may be affected by tumors and the tumor microenvironment. In addition, as the tumor progresses, the molecular level changes related to the characteristics of stem cells are a dynamic development process, and the stem cell score of INVASIVE is the highest.

In addition, to reveal the molecular contribution to stem scores changes, 299 driver genes’ expression was examined. Compared with the normal group, some driver genes in the IPMA, IPMC, and INVASIVE groups were consistently upregulated, including KRAS, ERBB3, RUNX1, and ELF3. Other driver genes, such as SOX17, Bcl-2, and TBX3, were consistently downregulated (Figures 2D–F). These results provide a deeper explanation at the RNA level; that is, these driver genes play an important role in IPMN tumorigenesis.

We also estimated the correlation between the expression of the eleven common driver genes and the stem scores. All the upregulated genes were significantly positively correlated with the stem scores (Figure 2G), and all the downregulated genes were significantly negatively correlated with the stem scores (Supplementary Figure S2). These results indicated that these genes are important in the dedifferentiated oncogenic phenotype of IPMN.

### The immune microenvironment is highly inactive in tumors

**FIGURE 3:**
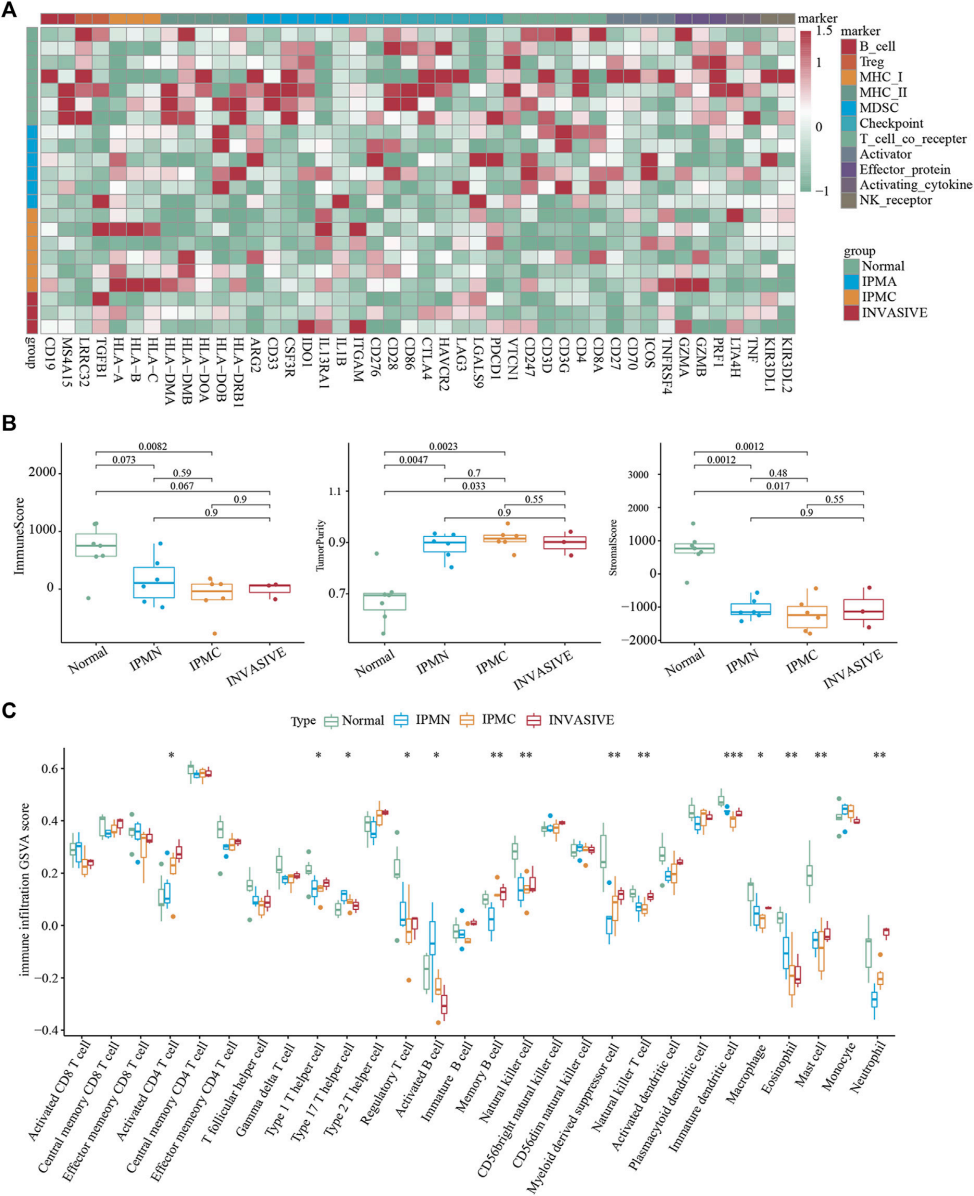
Immune microenvironment evaluation of lesions. **(A)** Unsupervised hierarchical clustering of Normal, IPMA, IPMC, and invasive samples using 46 immune cell markers. **(B)** Evaluation of Estimate, Immune, and Stromal Score. **(C)** The ssGSEA-inferred absolute infiltration of 28 immune cell types.*p < 0.05; **p < 0.01; ***p < 0.001. Boxplots show mean ± SEM.

To understand the changes in the immune microenvironment during the progression of IPMN, we first applied the immune infiltration scoring algorithm and drew a heatmap to show the overall level and changes in immune infiltration (Cancer Genome Atlas Research Network, 2017). As shown in Figure 3A, the immune score of the normal group was significantly higher than that of the other three groups, and the invasive group had the lowest immune score, which was consistent with the “cold tumor” characteristics of pancreatic cancer (Ajina and Weiner, 2020). The ESTIMATE algorithm also verified our results (Figure 3B). However, the immune scores between the IPMA, IPMC, and invasive groups were not significantly different (Figure 3B). Moreover, the ssGSEA algorithm was utilized to calculate further the relative infiltration abundance of 28 immune cell types. A significant increase in activated CD4 T cells, type 1 helper cells, memory B cells, myeloid-derived suppressor cells, and neutrophils was observed during the progression of IPMN. Meanwhile, eosinophils, activated B cells, and helper 17 cells displayed consistently reduced expression (Figure 3C).

### Macrophages and myeloid-derived cells are mainly involved in IPMN progression

**FIGURE 4:**
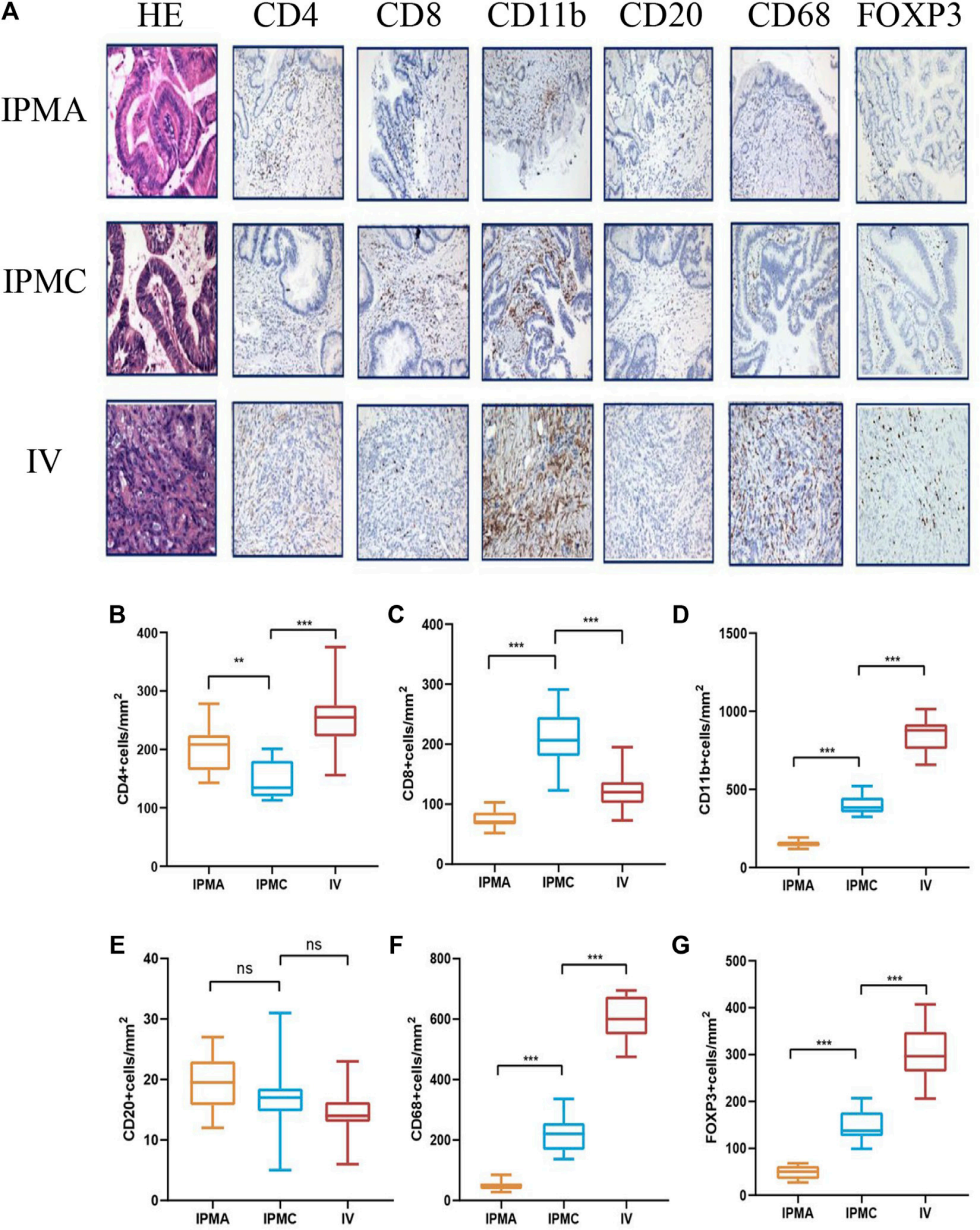
Representative HE and IHC images of different disease stage tissues. **(A)** From left to right, immunohistochemical staining for CD4, CD8, CD11b, CD20, CD68, and FOXP3. **(B–G)** Histogram showing the density of infiltrating lymphocytes.

To identify the specific immune cell types involved in IPMN progression, CD20 (B cells), CD4 (helper T cells), CD8 (cytotoxic T cells), Foxp3 (Treg cells), CD68 (macrophages), and CD11b (myeloid-derived cells), positive cells were analyzed by immunohistochemistry. As shown in Figure 4A, myeloid-derived cells and macrophages are the most prominent immune cells accumulating during IPMN progression. Moreover, their density is significantly higher than that of T cells and B cells, suggesting that these two types of cells may primarily be involved in the composition of the IPMN immune microenvironment (Figures 4D,F).

Myeloid-derived cells include many cell types; we mainly focused on the special type named myeloid-derived suppressor cells (MDSCs). In recent years, many studies have suggested that MDSCs play an important role in pancreatic cancer progression (Bernard et al., 2019; Fan et al., 2020). Since human myeloid-derived suppressor cells have an immunophenotype CD11b^+^CD33^+^HLA-DR^−^ (Gabrilovich and Nagaraj, 2009), we supplemented the immunohistochemistry with CD33 and HLA-DR antibodies, and our results confirmed the presence of MDSCs in IPMN tissues. CD11b^+^ cells expressed CD33 simultaneously, whereas HLA-DR was negative (Supplementary Figures S3A–C). Moreover, MDSCs can be divided into two groups: polymorphonuclear MDSCs (PMN-MDSCs) and mononuclear MDSCs (M-MDSCs) (Gabrilovich and Nagaraj, 2009). To identify the subtypes of MDSC, we further performed CD15 and CD14 staining on CD11b^+^ tissues. The results showed that these CD11b^+^ cells also expressed CD14 markers, suggesting that the most represented myeloid-derived cells that infiltrate during the progression of IPMN are monocytic (M) MDSCs (Supplementary Figures S3D–F). Macrophages also include subtypes M1 and M2, and further research is needed to distinguish the specific type that infiltrates around the tumor. Other common immune cells, such as CD4^+^ T cells, decreased to a certain extent during the IPMC stage. However, their expression increased significantly in the INVASIVE stage (Figure 4B), and their invasion mechanism needs further exploration. Unlike CD4^+^ T cells, CD8^+^ T cells showed a certain increase in the IPMC stage, but their expression was significantly reduced in the invasive group (Figure 4C). This is consistent with previous reports in pancreatic cancer that killer T cells cannot enter and have a killing effect when they reach the tumor periphery. Consistent with previous research reports, our results suggest that the number of regulatory T cells (Treg) gradually increases with disease progression (Figure 4G), suggesting the gradual formation of an immunosuppressive microenvironment. Unlike other immune cells, we could only see very few CD20^+^ B cells (<20 cells/mm^2^) in the components of IPMN at all stages, suggesting that B cells may be less involved in the formation of the immune microenvironment of IPMN (Figure 4E).

### Consistent DEGs play critical roles in IPMN tumorigenesis and progression

**FIGURE 5:**
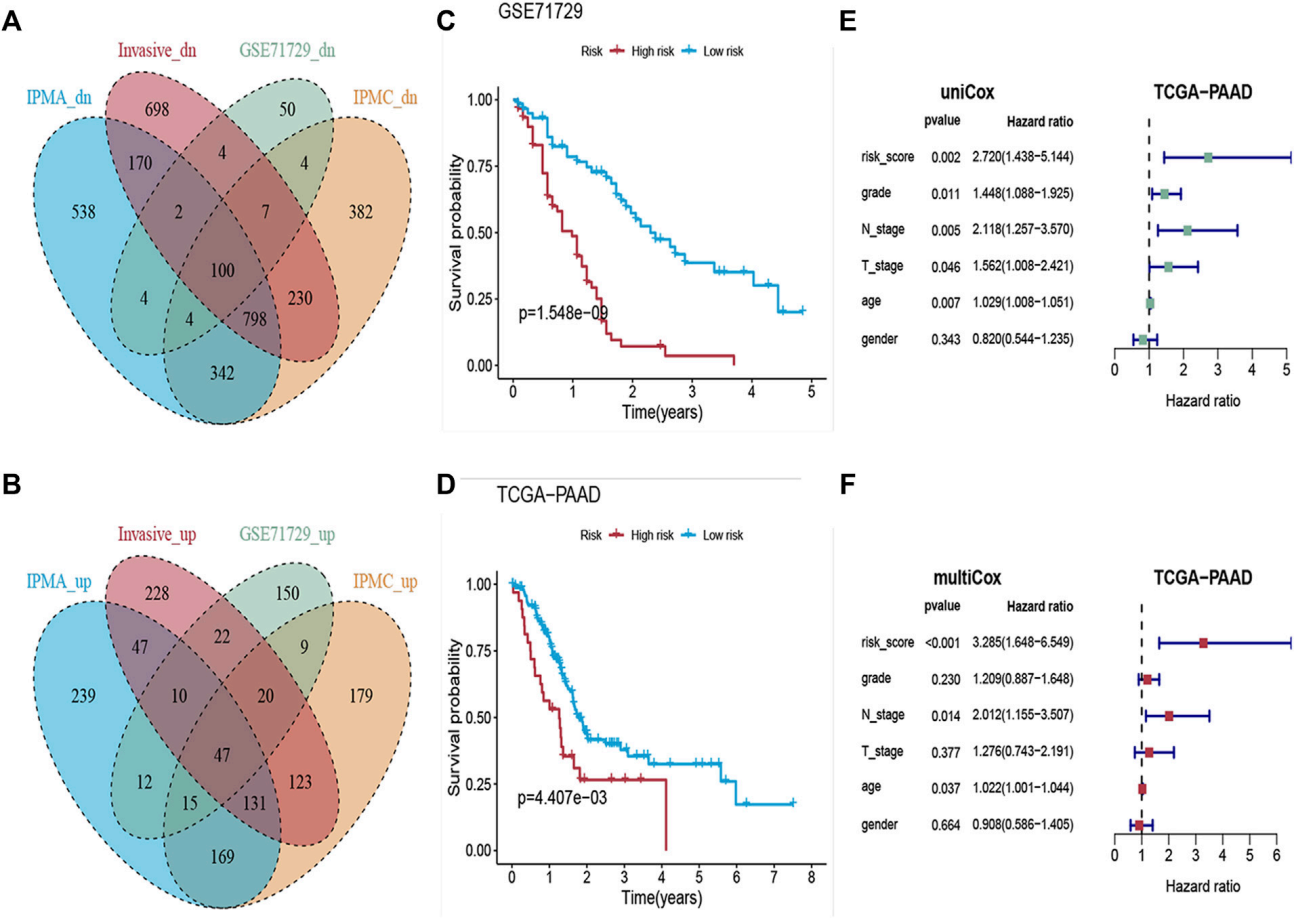
Construction of the Risk-Scoring Model. **(A,B)** Venn diagram showing the co-upregulated and co-downregulated genes in IPMA, IPMC, invasive and PDAC samples. **(C,D)** Kaplan-Meier analysis for overall survival according to the eleven-gene signature risk score in GSE71729 and TCGA cohorts. **(E,F)** Forest plots of the hazard ratios (HRs) with 95% confidence interval (95% CI) and *p*-value of univariate and multivariate Cox regression.

GSE71729 was used as a source of PDAC samples in this study, which included 145 primary PDAC samples and 46 normal pancreatic tissue samples. As shown in Figures 5A,B, there were 47 co-upregulated and 100 co-downregulated DEGs (co-up DEGs and co-down DEGs) in all four stages (IPMA, IPMC, INVASIVE, and PDAC). All co-up DEGs were significantly positively correlated with the stem scores, and all co-down DEGs were significantly negatively correlated with the stem scores (|cor| > 0.35, *p* value < 0.01, Supplementary Table S1). Given that these coDEGs continue to be dysregulated during IPMN tumorigenesis and progression, we wondered about their prognostic value. To detect the prognostic value of these 147 coDEGs, the GSE71729 dataset was designated as the training dataset. The LASSO Cox regression model narrowed the 147 coDEGs to eleven genes as the most useful prognostic markers (KCNK1, FHL2, LAMC2, CDCA7, GPX3, C7, VIP, HBA1, BTG2, MT1E, LYVE1).

We then constructed an eleven-gene signature and derived a formula to calculate the survival risk score for each patient based on the expression value of the eleven genes and weighted by the regression coefficient in the training dataset. We divided patients into high-risk or low-risk groups using the optimum cutoff score (cutoff = 2.58) obtained by the X-tile plot. Patients with lower risk scores had a better OS (n = 123, log-rank: *p* = 1.548e−09, Figure 5C) than patients with higher risk scores.

To further validate the prognostic value of the eleven-gene signature, we used the same formula and the same cutoff (cutoff = 2.58) to dichotomize TCGA PAAD patients (independent testing dataset) into high- or low-risk groups. Similar to the findings from the training set, patients in the high-risk group had a shorter median OS (log-rank: *p* = 4.407e−03, Figure 5D) than patients in the low-risk group. Univariate and multivariate Cox proportional hazard regression analyses showed that the prognostic capacities of the eleven-gene signature in OS had good prediction ability (Figures 5E,F).

### Important transcription factors in IPMN tumorigenesis and progression

**FIGURE 6:**
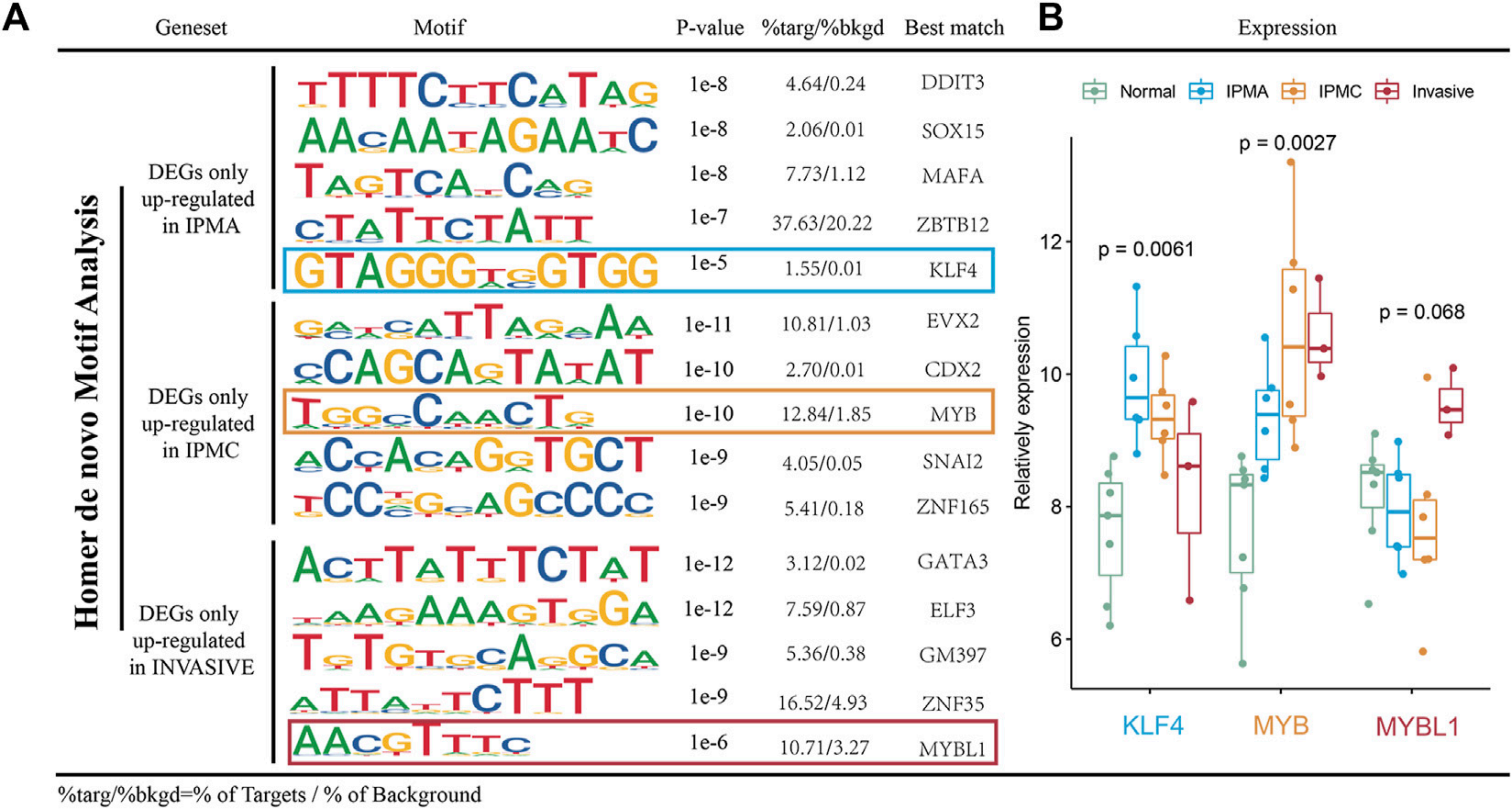
Motif analysis of DEGs. **(A)** Top 5 enriched known motifs as predicted *via* Homer analysis in different stages. **(B)** Relative mRNA expression of key TFs in different disease stage tissues.

Transcription factors (TFs) are evidently involved in a wide range of cellular functions, including activation, repression, and gene regulation (Lambert et al., 2018). Numerous transcription factors have been identified as regulating pancreatic cancer growth, including SMAD3 (Yamazaki et al., 2014) and TWIST1 (Wang et al., 2020), among others. However, TF regulatory mechanisms are rarely studied in the context of IPMN. To clarify whether some transcription factors can regulate the gene sets we found in different IPMN stages, we performed MOTIF analysis using HOMER software. Figure 6A illustrates the enriched motifs at different stages as well as the transcription factors that might bind to them. In the IPMA stage, genes are regulated by transcription factors like DDIT3, SOX15, MAFA, ZBTB12, and KLF4, while in the IPMC stage they are regulated by transcription factors like EVX2, CDX2, MYB, SNAI2, and ZNF165. As the invasive stage progressed, transcription factors like GATA3, ELF3, GM397, ZNF35, and MYBL1 controlled gene expression. We further investigated the indicated transcription factor mRNA expression levels across the four stages. According to Figure 6B, the mRNA levels of KLF4 were significantly higher in the IPMA stage than in the other three stages, suggesting that KLF4 mainly regulates gene expression in IPMA. Consistently, we found a high expression of MYB and MYBL1 genes in the IPMC and invasive stages, suggesting an important role of the MYB family in IPMN progression. Further mechanism research is needed to confirm our conclusion.

## Discussion

In recent years, the pathogenesis of IPMN has become an active area of research because it represents a definitive precancerous lesion of pancreatic cancer. Currently, most research has focused on detecting malignant tumors before surgery and deciding when surgery is needed (Maker et al., 2011; Basturk et al., 2015). However, little research has been conducted on how IPMN progresses from the normal ductal epithelium to early invasive tumors, as well as how it escapes immune system attack. In this study, we explored the possible pathogenesis of IPMN by analyzing the gene expression profiles of the epithelial components of IPMN at different developmental stages; in addition, we examined the infiltrating populations of immune cells within IPMN at different developmental stages to reveal why the immunosuppressive environment develops; finally, we identified a set of 11 genes that can be used to discriminate malignant IPMN, and that are closely associated with pancreatic cancer prognosis.

Differential gene enrichment analysis revealed that glycosylated proteins were upregulated in the early stage of disease development (IPMA). Glycosylation is a major posttranscriptional protein modification that plays an important role in many biological processes (Anguizola et al., 2013). Aberrant glycosylation is a universal feature of cancer cells, and it is well established that even small changes to the glycome can severely affect tumor cell biology (Costa et al., 2020). Consistent with our research results, numerous studies have suggested that glycosylated proteins such as CEA (You et al., 2016), CA19-9 (Ciprani et al., 2020), B7.1 (Aronsson et al., 2018), and MUC7 (Carrara et al., 2011) are related to IPMN progression, therefore supporting the idea of glycosylated proteins as early diagnostic indicators of IPMN. Following this, the epithelial cell proliferation ability was markedly enhanced (IPMC stage), which was consistent with the morphological observations and confirmed in the stem cell score analysis. Lastly, adhesion-related and ECM-receptor interaction pathways changed significantly from IPMC to invasive carcinoma, supporting an increased capacity for metastasis.

The role of driver gene mutations in IPMN development has been extensively studied. KRAS mutations are found in nearly half of IPMN patients, but KRAS mutations alone cannot lead to IPMN (Collet et al., 2020). GNAS gene mutation may be a specific gene mutation in IPMN patients (Hosoda et al., 2015). Other mutations, such as RNF43 (Sakamoto et al., 2015), TP53 (Noe et al., 2020), and SMAD4 (Noe et al., 2020), have also been reported. We examined the expression profiles of 299 ubiquitous driver genes in IPMN development and found consistent differentially expressed drivers, including KRAS, ERBB3, RUNX1, and ELF3, from IPMA to invasive cancer. ERBB3 (also known as HER3; human epidermal growth factor receptor 3) is a member of the epidermal growth factor receptor family (EGFR) that activates multiple downstream pathways (including MAPK/ERK and PI3K/AKT) to affect cell proliferation and differentiation (Holbro et al., 2003). It has been reported in many tumor types including breast, lung, and colorectal cancer (Sithanandam and Anderson, 2008; Hafeez et al., 2020). RUNX1, also known as acute myeloid leukemia 1 (AML1) protein, is a transcription factor that consists of 453 amino acids and modulates the differentiation of hematopoietic stem cells into functional blood cells (Simon et al., 2020). It is inactivated in patients with myelodysplasia and nonhereditary acute myelogenous leukemia. In recent years, emerging functions of RUNX1 in solid tumors have been shown (Riggio and Blyth, 2017). In pancreatic cancer, RUNX1 expression is upregulated and negatively correlated with patient prognosis (Liu et al., 2020). These two genes may play an important role in developing IPMN, and other mutation drivers may be altered to maintain the balance of biological functions. Our analysis of the mutational spectrum further enriches the understanding of IPMN pathogenesis, and more studies are needed to confirm our findings.

IPMN can progress from adenoma to invasive cancer, but the changes in the immune microenvironment during this process are still unclear. Some authors tried to analyze the specific changes in this process in previous studies. For example, Bernard’s single-cell sequencing study found that low-grade IPMN mainly manifested a pro-inflammatory state. With disease progression, the pro-inflammatory state is gradually suppressed, accompanied by an increase in myeloid-derived suppressor cells. Roth et al. focused on T cell subsets during IPMN progression, showing that T cells in low-grade IPMN became more heterogeneous, mainly composed of CD8^+^ T cells and CD4^+^ T cells. Although the Treg cells are relatively small, their number in invasive cancer significantly increases, constituting the immunosuppressive microenvironment’s main component. Our data analysis and immunohistochemical results suggest that during IPMN progression, myeloid cells and macrophages suffered the most drastic change, and their cell density is much higher than that of T cells and B cells, indicating a primary role in immunosuppression. The immunosuppressive role of myeloid cells by significantly inhibiting T cells’ function has been recently described (Serafini et al., 2008; Raber et al., 2012). To our knowledge, at the organizational level, we verified the accumulation of MDSCs in IPMN for the first time; At the same time, we also detected CD14 and CD15 of CD11b^+^ cells. The results indicated that the most obvious infiltration cells in IPMN progression were monocytic (M) MDSCs. Some treatment methods for MDSC have achieved initial results in many tumors. Based on our experimental results, we speculate that the treatment methods targeting MDSC may be applied to IPMN patients, especially in the IPMC stage. The current recommendation for IPMC patients is regular observation. However, it would be interesting to see if the tumor microenvironment could be altered and if IPMC patients could be classified by infiltration. Our study also found that macrophages and T-reg cells increased significantly in the progression process, accompanied by a decrease in CD8^+^ T cells, consistent with previous results (Di Federico et al., 2021). The complex tumor microenvironment is an important direction of tumor therapy. Our research results may have some suggestive significance. Additional large sample studies are needed to confirm our research results.

LASSO modeling was used to develop an 11-gene risk stratification model for identifying high-risk IPMN patients. This could facilitate appropriate clinical decision-making and improve the treatment outcome for such patients if the genes are detected early. Currently, the diagnosis of malignant IPMN is primarily based on imaging parameters and cyst fluid cytology results (Carr et al., 2018; Ciprani et al., 2020). The morphology of a cyst is usually nonspecific, while cytological analysis results can be affected by other factors, such as concomitant pancreatitis and other pancreatic cystic diseases (Yang et al., 2019). EUS-guided through-the-needle biopsies of cyst walls are widely used in clinical practice today, and their safety and reliability have been established (Mohammad Alizadeh et al., 2016; Kovacevic et al., 2020). Using DNA sequencing and chromogenic *in situ* hybridization (CISH) on needle biopsies could be useful in stratifying patients with IPMN and identifying those who require further treatment.

From a functional perspective, many of the genes identified in our biomarker panel may contribute to cancer pathogenesis. For example, Laminin subunit gamma-2 (LAMC2), a protein found in the epithelial basement membrane that controls cell motility and adhesion, is widely expressed in most human tumors (Wang et al., 2021). High LAMC2 expression in pancreatic cancer defines a highly metastatic stem cell subset with a reduced survival probability (Yang et al., 2020). Similarly, the potassium channel protein KCNK1, which has a two-hole domain (K2P), is dramatically increased in breast cancer tissues and is linked to a poor prognosis (Zou et al., 2022).

Transcriptional regulation plays a crucial role in tumor development, and this has been confirmed in many studies (Schuster et al., 2020; Vallejo et al., 2021). However, little is known about how transcriptional regulation regulates IPMN progression. Using expression profiling data for IPMN progression, we identified highly expressed genes at its different stages. Transcriptional regulatory sequences and transcription factors potentially involved in IPMN development are illustrated in Figure 6. HOMER prediction results and mRNA expression analysis reveal that KLF4 regulates gene expression mainly during the IPMA stage, while MYB and MYBL1 are involved during the IPMC and invasive stages. MYB, which is part of an important oncogene family, has received extensive attention for its relevance to various tumors (Ciciro and Sala, 2021), but its role in the development of IPMN has not been fully explained. Our data analysis results need to be validated by more experiments.

There are still some flaws in our study. First, our data came from public databases, and the number of samples in the INVASIVE group was relatively small, which might hide some positive results. In addition, our observations remain in the observation stage, therefore requiring further exploration of the IPMN development mechanism. Finally, IPMN is divided into four subtypes, gastric, intestinal, pancreatic bile duct, and eosinophilic type, of which eosinophilic type has been listed separately. The specific pathogenesis of the four subtypes may be different, and the prognosis is also different. For example, the gastric type mainly develops into tubular adenocarcinoma with a poor prognosis, while the intestinal type mainly develops into colloid carcinoma with a relatively good prognosis. Whether our prediction model applies to all patients needs further study.

In conclusion, our data indicated that within the IPMN progression, the stemness score of the tumor is continuously enhanced, and an immunosuppressive microenvironment is gradually developed. Therefore, the prognosis of these patients may be effectively improved if the tumors are treated at an early stage. Additionally, our constructed 11-gene risk dataset can effectively predict malignant IPMN patients and is closely related to the prognosis of pancreatic cancer patients. In summary, our study may be of great significance to the understanding of IPMN. Large samples or prospective studies will be needed to verify our conclusions in the future.

## Data Availability

The original contributions presented in the study are included in the article/Supplementary Material, further inquiries can be directed to the corresponding authors.

## Abbreviations

IPMN, intraductal papillary mucinous neoplasm; IPMA, intraductal papillary-mucinous adenoma; IPMC, intraductal papillary-mucinous carcinoma; PAAD, pancreatic adenocarcinoma; TCGA, The Cancer Genome Atlas; GEO, Gene Expression Omnibus; IHC, Immunohistochemistry; DEG, differentially expressed genes; GO, Gene ontology; KEGG, Kyoto Encyclopedia of Genes and Genomes; GSEA, Kyoto Encyclopedia of Genes and Genomes; LASSO, The least absolute shrinkage and selection operator; MDSC, Myeloid-derived suppressor cells; FC, Fold-change.
