# Supplementary material for: The molecular, immune features, and risk score construction of intraductal papillary mucinous neoplasm patients
Source: Front Mol Biosci. 2022 Aug 26;9:887887. doi: 10.3389/fmolb.2022.887887 (PMC9459388; doi:10.3389/fmolb.2022.887887)
Supplement: Supplementary file 1 [file Table1.DOCX]

Table S1. Correlation of co-expressed genes with stemness score.

| ID | Co_DEG.gene | Cor | Pvalue |
| --- | --- | --- | --- |
| 1 | C7 | -0.923207 | 4.08E-06 |
| 2 | HOMER2 | -0.858837 | 1.32E-06 |
| 3 | CD36 | -0.852061 | 1.04E-06 |
| 4 | GPX3 | -0.839639 | 9.01E-07 |
| 5 | SST | -0.83738 | 9.56E-07 |
| 6 | C6 | -0.826087 | 1.79E-06 |
| 7 | SCGN | -0.809376 | 5.07E-06 |
| 8 | PRSS1 | -0.800113 | 9.82E-06 |
| 9 | PDK4 | -0.800113 | 9.82E-06 |
| 10 | AMY1C | -0.783173 | 2.35E-05 |
| 11 | IAPP | -0.783173 | 2.35E-05 |
| 12 | PLA2G1B | -0.777527 | 3.04E-05 |
| 13 | FAM150B | -0.776398 | 3.19E-05 |
| 14 | GUCA1C | -0.763975 | 5.34E-05 |
| 15 | DPEP1 | -0.761717 | 5.83E-05 |
| 16 | RGN | -0.759458 | 6.36E-05 |
| 17 | PDIA2 | -0.752682 | 8.17E-05 |
| 18 | GP2 | -0.751553 | 8.51E-05 |
| 19 | NPTX2 | -0.750423 | 8.86E-05 |
| 20 | ZBTB16 | -0.750423 | 8.86E-05 |
| 21 | GC | -0.747036 | 9.97E-05 |
| 22 | DPT | -0.744777 | 0.000108 |
| 23 | SLC4A4 | -0.742518 | 0.000116 |
| 24 | KCNJ16 | -0.73913 | 0.00013 |
| 25 | GSTA1 | -0.738001 | 0.000135 |
| 26 | ERO1LB | -0.736872 | 0.00014 |
| 27 | CPB1 | -0.722191 | 0.000221 |
| 28 | CLEC3B | -0.722191 | 0.000221 |
| 29 | VIP | -0.722191 | 0.000221 |
| 30 | GATM | -0.717674 | 0.000253 |
| 31 | REG3G | -0.712027 | 0.000297 |
| 32 | CXCL12 | -0.710898 | 0.000307 |
| 33 | AOX1 | -0.700734 | 0.000406 |
| 34 | AMBP | -0.692829 | 0.0005 |
| 35 | PNLIPRP1 | -0.689441 | 0.000545 |
| 36 | PAIP2B | -0.686053 | 0.000594 |
| 37 | FLRT2 | -0.681536 | 0.000665 |
| 38 | SERPINI2 | -0.67721 | 0.000536 |
| 39 | TPST2 | -0.677019 | 0.000742 |
| 40 | CPA1 | -0.671372 | 0.00085 |
| 41 | PNLIP | -0.669113 | 0.000896 |
| 42 | BEX1 | -0.666855 | 0.000945 |
| 43 | SYCN | -0.663467 | 0.001022 |
| 44 | CUZD1 | -0.652174 | 0.001317 |
| 45 | CTRB1 | -0.648786 | 0.001419 |
| 46 | NPY | -0.644269 | 0.001564 |
| 47 | AQP1 | -0.64201 | 0.001642 |
| 48 | RBP1 | -0.640881 | 0.001681 |
| 49 | CTRC | -0.639752 | 0.001722 |
| 50 | TRPV6 | -0.637493 | 0.001806 |
| 51 | INS | -0.632976 | 0.001984 |
| 52 | ERP27 | -0.629588 | 0.002126 |
| 53 | SV2B | -0.629588 | 0.002126 |
| 54 | CTRL | -0.627329 | 0.002226 |
| 55 | CTRB2 | -0.625071 | 0.00233 |
| 56 | FIGF | -0.625071 | 0.00233 |
| 57 | TMED6 | -0.621683 | 0.002493 |
| 58 | REG1A | -0.621683 | 0.002493 |
| 59 | FAM107A | -0.620553 | 0.00255 |
| 60 | AKAP7 | -0.616036 | 0.002787 |
| 61 | BTG2 | -0.613778 | 0.002912 |
| 62 | CPA2 | -0.60926 | 0.003176 |
| 63 | GRB14 | -0.607002 | 0.003316 |
| 64 | ADARB2 | -0.603614 | 0.003535 |
| 65 | CLPS | -0.602484 | 0.003611 |
| 66 | CRYAB | -0.601355 | 0.003688 |
| 67 | HBA1 | -0.599097 | 0.003846 |
| 68 | HBA2 | -0.599097 | 0.003846 |
| 69 | FXYD2 | -0.597967 | 0.003927 |
| 70 | KIRREL2 | -0.595709 | 0.004094 |
| 71 | GAMT | -0.588933 | 0.004629 |
| 72 | PAH | -0.588933 | 0.004629 |
| 73 | PNLIPRP2 | -0.587804 | 0.004724 |
| 74 | AQP8 | -0.587804 | 0.004724 |
| 75 | CEL | -0.58684 | 0.004092 |
| 76 | TEX11 | -0.583286 | 0.00512 |
| 77 | REG1P | -0.581028 | 0.005328 |
| 78 | LYVE1 | -0.57651 | 0.005764 |
| 79 | MT1M | -0.575381 | 0.005878 |
| 80 | CA4 | -0.570864 | 0.006352 |
| 81 | MT1E | -0.562959 | 0.007258 |
| 82 | PRSS2 | -0.551666 | 0.008737 |
| 83 | SLC39A5 | -0.537136 | 0.009944 |
| 84 | LMO3 | -0.535855 | 0.011221 |
| 85 | LGALS2 | -0.534726 | 0.011419 |
| 86 | SPACA3 | -0.52908 | 0.012451 |
| 87 | PPP1R1A | -0.52697 | 0.011738 |
| 88 | PRSS3 | -0.521174 | 0.014024 |
| 89 | SCGB3A1 | -0.520045 | 0.014261 |
| 90 | REG1B | -0.513269 | 0.015756 |
| 91 | ANPEP | -0.509881 | 0.016551 |
| 92 | FGL1 | -0.485037 | 0.023437 |
| 93 | GNMT | -0.48235 | 0.022997 |
| 94 | AQP12A | -0.473744 | 0.027255 |
| 95 | ATP4A | -0.457498 | 0.032287 |
| 96 | BNIP3 | -0.442123 | 0.040677 |
| 97 | REG3A | -0.434218 | 0.044741 |
| 98 | SLC43A1 | -0.396951 | 0.068408 |
| 99 | GSTA3 | -0.391304 | 0.072709 |
| 100 | KLK1 | -0.389046 | 0.074486 |
| 101 | PLA2G10 | 0.36646 | 0.094146 |
| 102 | AKR1B10 | 0.442123 | 0.040677 |
| 103 | LAMB3 | 0.452287 | 0.035888 |
| 104 | PPARG | 0.452287 | 0.035888 |
| 105 | GALNT5 | 0.465839 | 0.030216 |
| 106 | TSPAN1 | 0.466968 | 0.029778 |
| 107 | CAPN8 | 0.498588 | 0.01944 |
| 108 | MYEOV | 0.499718 | 0.019134 |
| 109 | CTSE | 0.505364 | 0.017661 |
| 110 | MLPH | 0.507623 | 0.017099 |
| 111 | BCAS1 | 0.521174 | 0.014024 |
| 112 | CLDN18 | 0.521174 | 0.014024 |
| 113 | CDCA7 | 0.523433 | 0.013558 |
| 114 | OCIAD2 | 0.533597 | 0.011619 |
| 115 | BIK | 0.538114 | 0.010834 |
| 116 | MBOAT2 | 0.542631 | 0.010093 |
| 117 | TFF1 | 0.543761 | 0.009915 |
| 118 | LIPH | 0.553924 | 0.008423 |
| 119 | S100P | 0.566347 | 0.006858 |
| 120 | KCNK1 | 0.57651 | 0.005764 |
| 121 | SLC6A14 | 0.582157 | 0.005223 |
| 122 | VILL | 0.587804 | 0.004724 |
| 123 | C19orf33 | 0.587804 | 0.004724 |
| 124 | GPRC5A | 0.596838 | 0.004009 |
| 125 | ST6GALNAC1 | 0.600226 | 0.003766 |
| 126 | NQO1 | 0.601355 | 0.003688 |
| 127 | TMPRSS4 | 0.611519 | 0.003042 |
| 128 | AGR2 | 0.612648 | 0.002976 |
| 129 | ADAM8 | 0.618295 | 0.002666 |
| 130 | C15orf48 | 0.620553 | 0.00255 |
| 131 | MAL2 | 0.636364 | 0.001849 |
| 132 | LAMC2 | 0.65782 | 0.001162 |
| 133 | PTK6 | 0.666855 | 0.000945 |
| 134 | C1GALT1 | 0.677019 | 0.000742 |
| 135 | PLAC8 | 0.686053 | 0.000594 |
| 136 | FUT3 | 0.689441 | 0.000545 |
| 137 | REG4 | 0.689441 | 0.000545 |
| 138 | FXYD3 | 0.697346 | 0.000444 |
| 139 | FHL2 | 0.718803 | 0.000245 |
| 140 | SERPINB5 | 0.736872 | 0.00014 |
| 141 | NUF2 | 0.741389 | 0.000121 |
| 142 | NR1I2 | 0.75607 | 7.22E-05 |
| 143 | NMU | 0.760587 | 6.09E-05 |
| 144 | CEACAM5 | 0.806889 | 6.57E-06 |
| 145 | ATP2C2 | 0.808018 | 6.12E-06 |
| 146 | SDR16C5 | 0.811406 | 4.93E-06 |
| 147 | SLC26A9 | 0.823828 | 2.09E-06 |
