# Supplementary figures and images for: The molecular, immune features, and risk score construction of intraductal papillary mucinous neoplasm patients
Source: Front Mol Biosci. 2022 Aug 26;9:887887. doi: 10.3389/fmolb.2022.887887 (PMC9459388; doi:10.3389/fmolb.2022.887887)

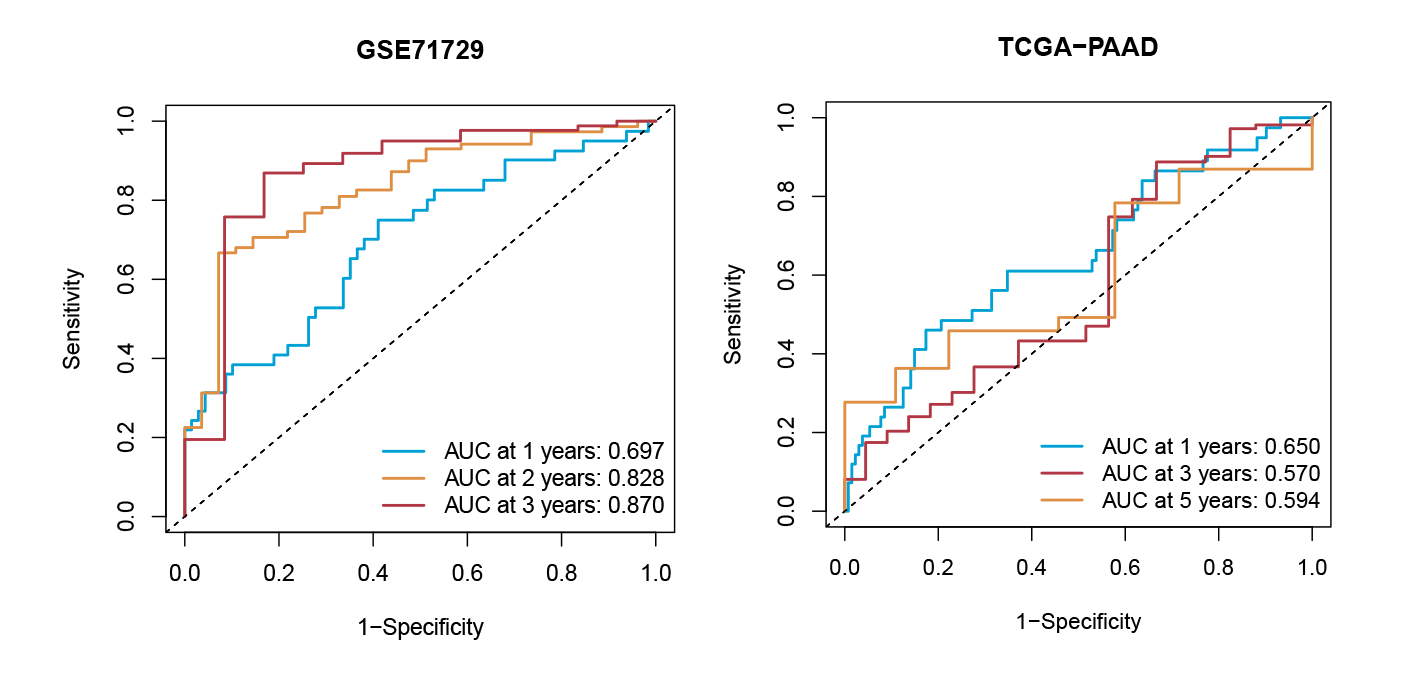

Supplement: Supplementary file 2 [file Image3.TIF]

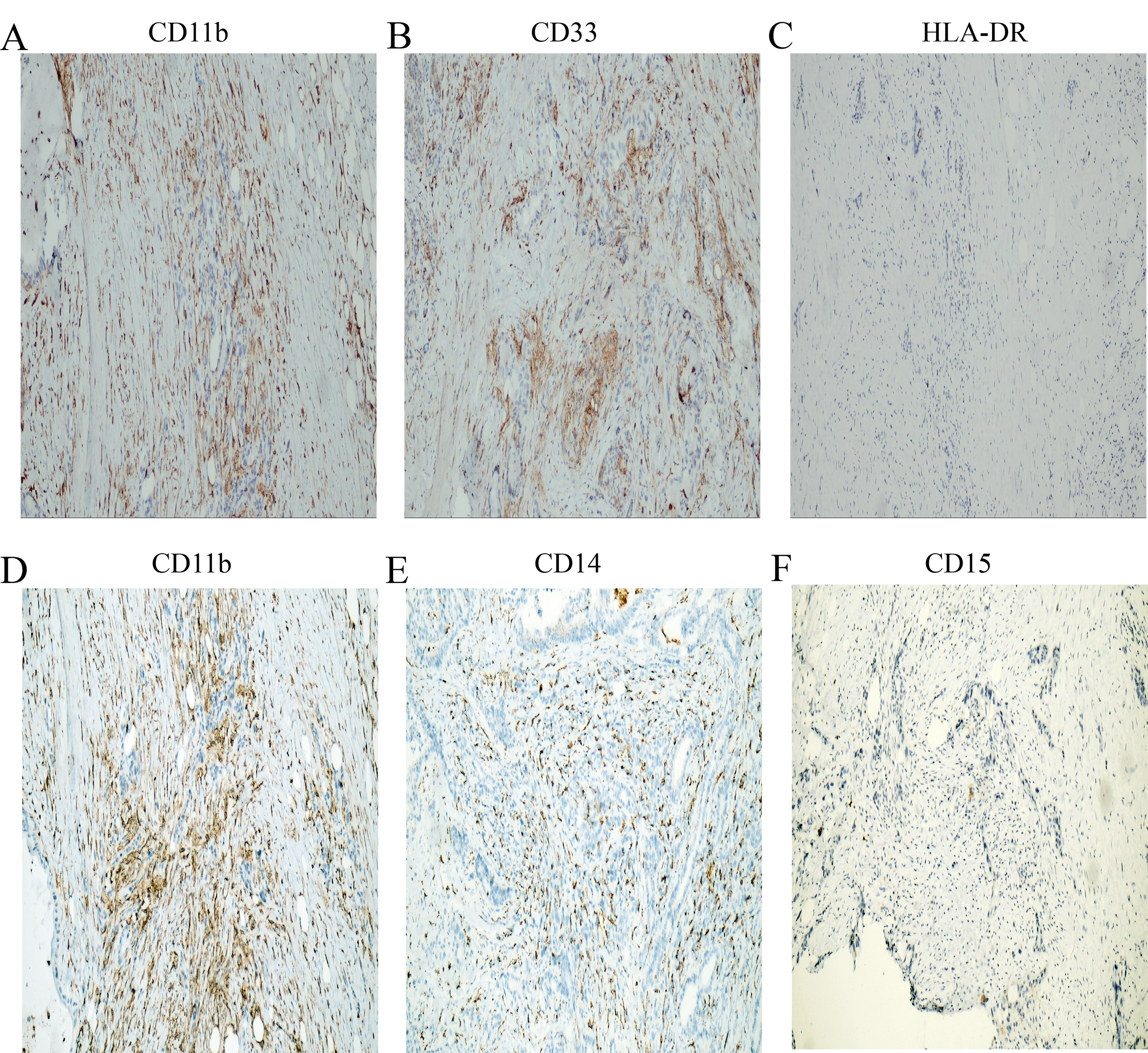

Supplement: Supplementary file 3 [file Image4.TIF]

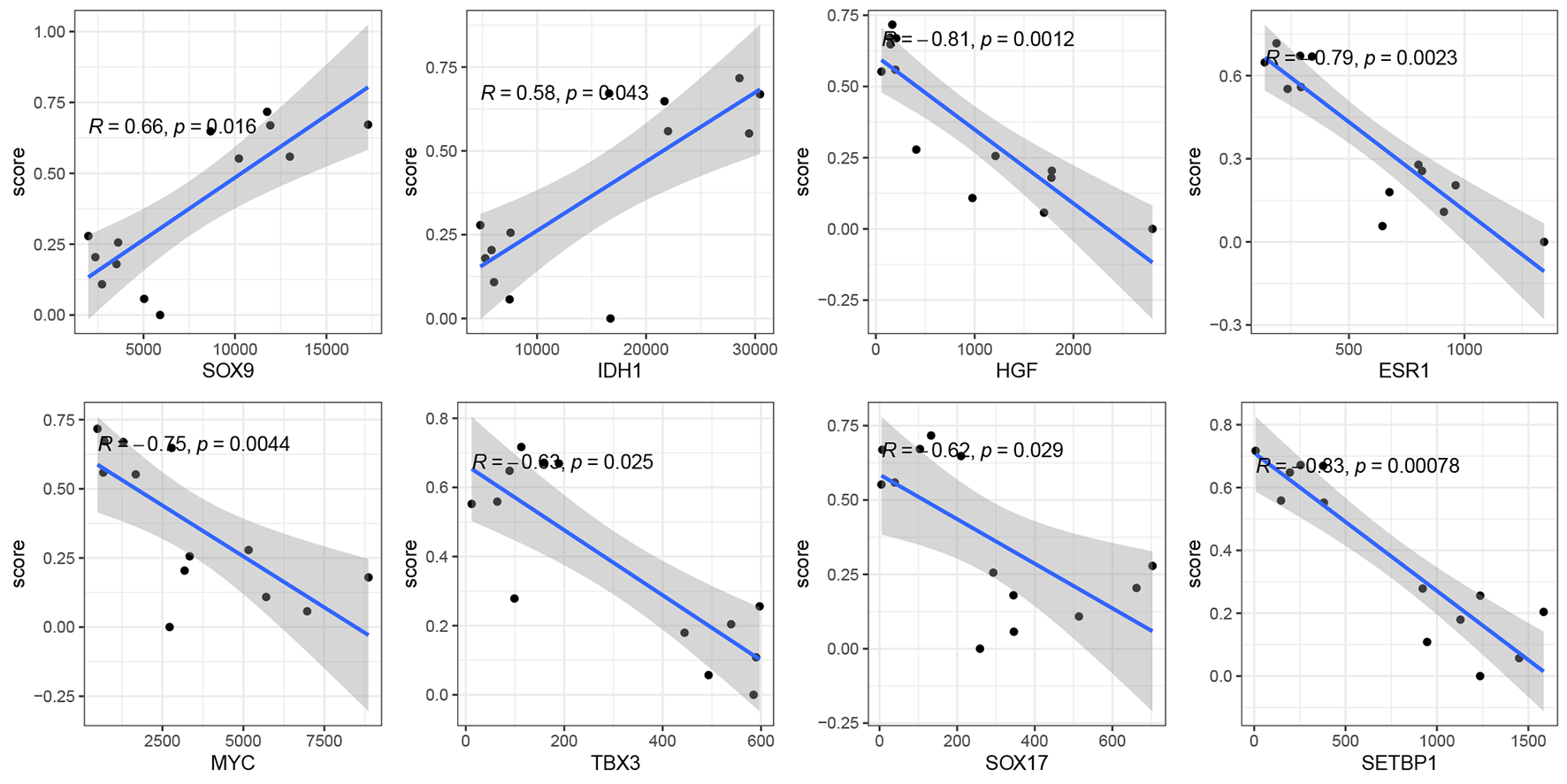

Supplement: Supplementary file 4 [file Image2.TIF]

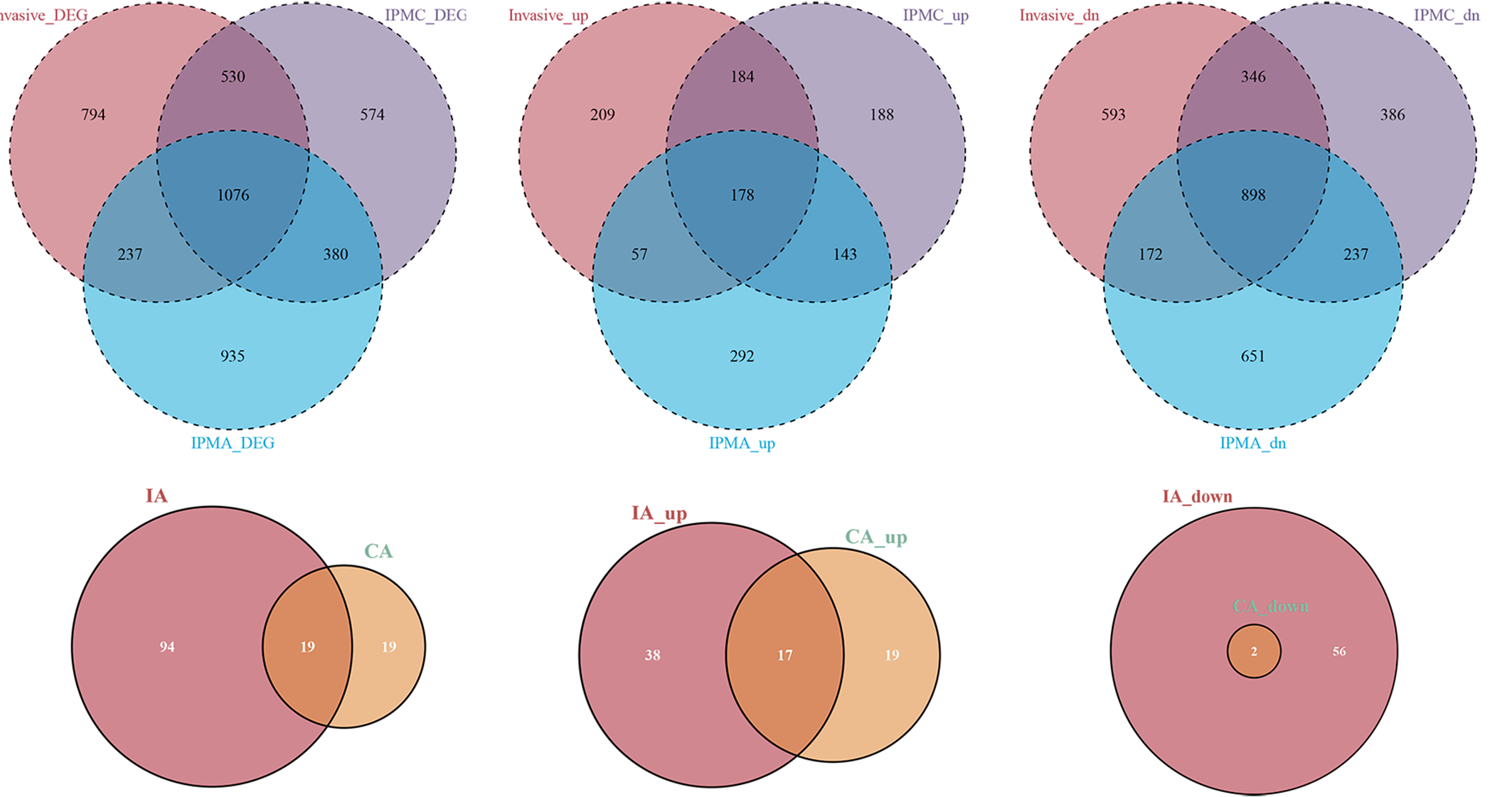

Supplement: Supplementary file 5 [file Image1.TIF]
