# Supplementary material for: The molecular, immune features, and risk score construction of intraductal papillary mucinous neoplasm patients
Source: Front Mol Biosci. 2022 Aug 26;9:887887. doi: 10.3389/fmolb.2022.887887 (PMC9459388; doi:10.3389/fmolb.2022.887887)
Supplement: Supplementary file 6 [file Table2.DOCX]

**Table S2** Description of the 3 datasets used in our study.

| Datasets | Sample type | Total  (n=) | Platform | Sample information |
| --- | --- | --- | --- | --- |
| GSE19650 | IPMN | 22 | Affymetrix Human Genome U133 Plus 2.0 Array | 22 normal and neoplastic epithelial cells from frozen tissue sections  (normal main pancreatic duct, IPMA, IPMC, and invasive carcinoma originating in IPMN). |
| GSE71729 | Pancreatic cancer | 340 | Agilent-014850 Whole Human Genome  Microarray 4x44K G4112F | 145 primary and 61 metastatic PAAD tumors, 17 cell lines, 46 pancreas and 88 distant site adjacent normal samples |
| TCGA-PAAD | Pancreatic cancer | 148 | Illumina HiSeq 2000 RNA sequencing | 148 patients with both  gene expression data and survival data |

IPMN: intraductal papillary mucinous neoplasm; TCGA: The Cancer Genome Atlas; PAAD: Pancreatic adenocarcinoma.
