# Supplementary material for: The molecular, immune features, and risk score construction of intraductal papillary mucinous neoplasm patients
Source: Front Mol Biosci. 2022 Aug 26;9:887887. doi: 10.3389/fmolb.2022.887887 (PMC9459388; doi:10.3389/fmolb.2022.887887)
Supplement: Supplementary file 7 [file Table3.DOCX]

Table S3. 46 immune genes used in analysis of immune microenvironment

| **Symbol** | **Markers** |
| --- | --- |
| CD19 | B_cell |
| MS4A15 | B_cell |
| LRRC32 | Treg |
| TGFB1 | Treg |
| HLA-A | MHC_I |
| HLA-B | MHC_I |
| HLA-C | MHC_I |
| HLA-DMA | MHC_II |
| HLA-DMB | MHC_II |
| HLA-DOA | MHC_II |
| HLA-DOB | MHC_II |
| HLA-DRB1 | MHC_II |
| ARG2 | MDSC |
| CD33 | MDSC |
| CSF3R | MDSC |
| IDO1 | MDSC |
| IL13RA1 | MDSC |
| IL1B | MDSC |
| ITGAM | Checkpoint |
| CD276 | Checkpoint |
| CD28 | Checkpoint |
| CD86 | Checkpoint |
| CTLA4 | Checkpoint |
| HAVCR2 | Checkpoint |
| LAG3 | Checkpoint |
| LGALS9 | Checkpoint |
| PDCD1 | Checkpoint |
| VTCN1 | T_cell_co_recepter |
| CD247 | T_cell_co_recepter |
| CD3D | T_cell_co_recepter |
| CD3G | T_cell_co_recepter |
| CD4 | T_cell_co_recepter |
| CD8A | T_cell_co_recepter |
| CD27 | Activator |
| CD70 | Activator |
| ICOS | Activator |
| TNFRSF4 | Activator |
| GZMA | Effector_protein |
| GZMB | Effector_protein |
| PRF1 | Effector_protein |
| LTA4H | Activating_cytokine |
| TNF | Activating_cytokine |
| KIR3DL1 | NK_receptor |
| KIR3DL2 | NK_receptor |
